# Spontaneous spinal CSF leaks: a rare variant exome sequencing study and functional analysis

**DOI:** 10.1016/S1474-4422(26)00140-7

**Published:** 2026-07

**Authors:** Cassie A Parks, Mukti Singh, Elizabeth Wohler, Renan Martin, Silke Peeters, Emily E Juzwiak, Xinyi Sun, Bart L Loeys, Nara Sobreira, Clair Baldock, Wouter I Schievink, Harry C Dietz

**Affiliations:** a Department of Genetics, Johns Hopkins Medicine, Baltimore, MD, USA; b Division of Cell-Matrix Biology and Regenerative Medicine, University of Manchester, Manchester, UK; c Center of Medical Genetics, University of Antwerp and Antwerp University Hospital, Antwerp, Belgium; d Department of Neurosurgery, Cedars-Sinai Medical Center, Los Angeles, CA, USA

## Abstract

**Background:**

Spontaneous spinal CSF leaks are associated with connective tissue diseases including Marfan syndrome and Loeys–Dietz syndrome, which are caused by mutations in genes that influence the content and integrity of the extracellular matrix. Patients with spontaneous spinal CSF leaks without a defined connective tissue disease diagnosis can show subtle or non-specific connective tissue disease manifestations, suggesting that mutations in extracellular matrix proteins might contribute to more common presentations of this condition. By doing a whole-exome sequencing study, we aimed to elucidate the genetic basis of spontaneous spinal CSF leaks.

**Methods:**

Through retrospective medical record review at Cedars-Sinai Hospital (Los Angeles, USA), we identified 42 individuals who had lateral spontaneous (ie, type 1b) spinal CSF leaks. We did a whole-exome sequencing study in these individuals and compared these data with the results of whole-exome sequencing for three independent control cohorts (2244 unrelated and unaffected adults recruited from various sites in the USA and 714 and 913 individuals recruited to separate sequencing initiatives at the University of Antwerp, Belgium). We used an in-silico prediction tool to establish the location of variants in the tertiary structure of the protein encoded by the top candidate gene. We also tested wild-type and mutant protein fragments for integrin-mediated binding to human dural fibroblasts in vitro. With CRISPR–Cas9 gene editing, we generated three mouse models harbouring different variants in the top candidate gene equivalent to variants found in individuals with type 1b spontaneous spinal CSF leaks and compared them with an established mouse model of Marfan syndrome. Intrathecal infusion testing was used to establish dural integrity and CSF leak properties in these mice.

**Findings:**

Whole exome sequencing on 42 unrelated individuals with type 1b spontaneous spinal CSF leaks who had been seen between Jan 1, 2006 and Dec 31, 2019 (35 [83%] were women, seven [17%] were men, 38 [90%] were White, two [5%] were Black, and two [5%] were Hispanic) identified potential causative genes. Nine (21%) of 42 individuals with type 1b spontaneous spinal CSF leak had rare functional variants in *FBN2*. Significant enrichment in rare functional *FBN2* variants was observed on comparison of the patient cohort with the Mendel discovery cohort (177 [8%] of 2244, p=0·041, odds ratio (OR) 3·18 [95% CI 1·50–6·76]) and the Belgian whole-exome (51 [7%] of 714, p=0·004, OR 3·55 [1·61–7·81]) and Belgian thoracic aortic aneurysm and dissection (45 [5%] of 913, p=0·0003, OR 5·26 [2·38–11·66]) validation cohorts. *FBN2* variants in individuals with type 1b spontaneous spinal CSF leaks showed a non-random domain distribution in fibrillin-2, with enrichment in TGF-β binding protein-like (TB) domains. Two out of three tested variants reduced fibrillin-2 fragment adhesion to human dural fibroblast in vitro. Mice carrying variants equivalent to the three *FBN2* variants in TB domains found in individuals with type 1b spontaneous spinal CSF leak (*Fbn2^A1052T/+^, Fbn2^D1581V/+^*, and *Fbn2^M2387T/+^*) showed a predisposition for dural rupture on controlled leak induction. Marfan syndrome mice (*Fbn1^C1039G/+^*) had increased meningeal compliance.

**Interpretation:**

Rare deleterious variants in *FBN2* might cause type 1b spontaneous spinal CSF leak, supporting the integration of *FBN2* genetic testing into clinical practice. As in other connective tissue diseases, the disruption of cell adhesion to extracellular matrix proteins might participate in the pathophysiology of spontaneous spinal CSF leaks. The generation of mouse models of spontaneous spinal CSF leaks will help the development of pharmacological therapeutic strategies.

**Funding:**

The Howard Hughes Medical Institute, the Marfan Foundation, the Pease–Scheeler Fund, and the Biotechnology and Biological Sciences Research Council.

## Introduction

Spontaneous spinal CSF leaks are an under-recognised cause of debilitating orthostatic headaches and neurological dysfunction. Epidemiological data indicate an annual incidence of 3·7 per 100 000 people, which is similar to that of trigeminal neuralgia.^1^ Patients have profound disability, with 75% reporting severe headache-related impairment and quality-of-life scores lower than those reported for patients with multiple sclerosis.^2^ Visualising spontaneous spinal CSF leaks often requires sophisticated and invasive imaging, and even with these spinal imaging techniques, clinicians frequently face diagnostic uncertainty. Available treatments present a challenging dilemma: epidural blood patching often provides only temporary relief, whereas surgical repair is not widely available and carries risk.^3^

**Research in context**

**Evidence before this study**

We searched PubMed from database inception to September, 2025 for original research and review articles, without language restrictions, using the search terms “spontaneous spinal CSF leak”, “spontaneous intracranial hypotension”, “fibrillin-2”, “FBN2”, and “type 1b CSF leak”. Before this work, it was known that individuals with specific systemic connective tissue disorders such as Marfan syndrome and Loeys–Dietz syndrome have an increased risk for spontaneous spinal CSF leaks, but no cause had been identified for non-syndromic spontaneous spinal CSF leaks.

**Added value of this study**

To our knowledge, this is the first study to define a genetic cause, specifically variants in *FBN2*, in the pathophysiology of spontaneous spinal CSF leaks. Functional in-vitro studies and animal model data are supportive of a causal effect of the identified variants in *FBN2*. The identified disease variants implicate impairment of integrin binding to fibrillin-2 as a relevant disease mechanism.

**Implications of all the available evidence**

Our findings are supportive of previous knowledge regarding a mechanistic role for impaired connective tissue integrity in the pathophysiology of spontaneous spinal CSF leaks. Novel contributions include specific implication of pathogenic variants in *FBN2*, the development of an animal model, and the application of a quantitative assay for dural biomechanical integrity. Taken together, these advancements will facilitate the development of refined diagnostic and treatment strategies.

The pathogenesis of spontaneous spinal CSF leaks is poorly understood. It involves structural defects in the dura mater, which is comprised primarily of collagen and elastin fibres, which provide biomechanical strength. Three types of spontaneous spinal CSF leaks have been identified on the basis of imaging and surgical data: ventral CSF leaks (ie, ventral to the spinal cord; type 1a), lateral CSF leaks (ie, lateral to the spinal cord; type 1b), CSF leaks associated with simple and complex meningeal diverticula (type 2a and type 2b, respectively), and CSF–venous fistulas (type 3).^4^ Although minor trauma precedes symptom onset in approximately one-third of patients,^3^ suggesting an environmental contribution to disease pathogenesis, mounting evidence indicates that genetic factors predisposing to connective tissue fragility play a central role in disease susceptibility.^5^, ^6^

Approximately two-thirds of patients with spontaneous spinal CSF leaks display clinical features of a generalised connective tissue disorder.^6^, ^7^ These clinical presentations range from established diagnoses such as Marfan syndrome, Loeys–Dietz syndrome, or Ehlers–Danlos syndrome to more subtle presentations including isolated arachnodactyly that do not meet formal syndromic diagnostic criteria.^7^, ^8^, ^9^ The observation that approximately 40% of patients with spontaneous spinal CSF leaks exhibit joint hypermobility and approximately 20% display Marfanoid skeletal features strongly suggests an underlying extracellular matrix defect.^5^, ^7^

Heritable disorders of connective tissue are frequently caused by mutations in genes encoding extracellular matrix proteins. Fibrillin-1 and fibrillin-2 are key components of microfibrils that provide scaffolding for elastic fibre assembly, a crucial component of the dura mater, and regulate growth factor signalling.^10^ Mutations in the genes encoding these proteins cause disorders with distinct phenotypes. *FBN1* mutations in calcium-binding epidermal growth factor (cbEGF)-like domains and some TGF-b binding protein-like (TB) domains cause Marfan syndrome, which is characterised by tall stature and aortic aneurysms.^11^ Contrastingly, mutations in and around the sole arginine–glycine–aspartic acid (RGD) sequence in fibrillin-1 cause stiff skin syndrome, which is characterised by skin fibrosis and short stature, and notably has no cardiovascular manifestations.^12^ Additional distinctive domain-specific phenotypes associated with *FBN1* mutations include geleophysic dysplasia, acromicric dysplasia and Weill–Marchesani syndrome.^11^ Additionally, mutations in cbEGF-like domains of *FBN2* cause congenital contractural arachnodactyly (Beals syndrome), which shares features with Marfan syndrome along with prominent joint contractures and scoliosis.^13^ This knowledge suggests that other domain-specific mutations in fibrillin-1 or fibrillin-2 could cause previously unidentified phenotypes.

Despite the association between spontaneous spinal CSF leaks and connective tissue disease characteristics, the genetic defects underlying spontaneous spinal cerebrospinal fluid leaks in the majority of patients have remained unknown. We aimed to understand the genetic basis of ssCSFLs, because deepening our understanding of its pathogenesis could transform diagnostic and therapeutic practices for this disabling condition.

## Methods

### Study design and participants

We focused on type 1b spontaneous spinal CSF leaks given the absence of an obvious anatomical predisposition, in contrast with other types (ie, bone spurs in type 1a, diverticula in type 2, or venous fistulae in type 3). We retrospectively identified patients with type 1b spontaneous spinal CSF leaks who had been seen as outpatients at Cedars-Sinai Medical Center, Los Angeles, CA, USA. Approval was obtained from the Cedars-Sinai institutional review board (Pro00049126). The inclusion criteria were CT or digital subtraction myelography-confirmed type 1b spontaneous spinal CSF leaks, no inciting trauma, and age younger than 45 years. Three control cohorts were used. The initial discovery cohort comprised 2244 admixed American unrelated adults who underwent whole-exome sequencing at the Baylor-Hopkins Center for Mendelian Genomics between January, 2012 and December, 2021 for an indication unrelated to spontaneous spinal CSF leak or a recognised systemic connective tissue disorder, as previously described.^14^ Two validation control cohorts of unrelated and unaffected individuals were ascertained by the Department of Medical Genetics at Antwerp University Hospital and subsequently analysed. One of these control cohorts comprised 913 Belgian individuals who underwent whole-exome sequencing owing to various cardiovascular indications without associated spontaneous spinal CSF leak (ie, the Belgian thoracic aortic aneurysm and dissection cohort). The other control cohort comprised 714 healthy parents of children with various presentations of intellectual disability as previously described (ie, the Belgian whole-exome cohort).^15^ All participants provided written informed consent for salivary sample collection for subsequent genetic analysis.

### Procedures

Daytime salivary samples were collected from individuals with type 1b spontaneous spinal CSF leaks by use of a DNA self-collection kit (Oragene**,** DNAGenotek, Canada). Samples were shipped at room temperature from Cedars-Sinai Hospital in Los Angeles, CA, USA to Johns Hopkins Medical Institutes in Baltimore, MD, where they were sequenced. Samples were stored for up to 9 months at room temperature or colder. We used the Perkin Elmer Chemagic MSM1 system (Johns Hopkins Fragment Analysis Facility) to extract DNA. Whole-exome sequencing was done with NovaSeq 6000 (Illumina, CA, USA; 100 base pair paired-end, S2 chemistry, error rate 0·01%). Data processing for the *GRCh37*/*hg19* genome was done by use of Burrows–Wheeler Alignment version 0.7.15,^16^ Genome Analysis Toolkit version 3.7 (Broad Institute, Cambridge, MA, USA), variant quality score recalibration to variant calling quality control, and the web-based portal PhenoDB to identify rare (minor allele frequency <0·01, Genome Aggregation Database [gnomAD] version 4.1.0) functional variants. To predict the deleteriousness of base substitution variants, we used the combined annotation dependent depletion scores (gnomAD version 4.1.0). Genes with more than 1000 mean transcript counts from a publicly available human cranial dura mater RNAseq dataset^17^ were considered expressed and the only genes explored in the rest of the study**.**

To assess the enrichment of specific alleles in individuals with type 1b spontaneous spinal CSF leaks, we compared the whole-exome sequencing data from the 42 patients with type 1b spontaneous spinal CSF leaks with that of the three independent control cohorts via gene burden analysis.

To assess the structural consequences of candidate deleterious variants, we generated in-silico models of fibrillin-2 TB domain fragments. We did in-vitro functional analyses on recombinant peptides to test their ability to bind to human dural fibroblasts. The detailed methods are provided in the appendix (p 1).

To assess the biomechanical consequences of candidate deleterious *FBN2* variants in vivo, we generated mice harbouring the corresponding *Fbn2* amino acid substitution in heterozygosity (*Fbn2*^A1052T/+^, *Fbn2*^D1581V/+^, and *Fbn2*^M2387T/+^). Mice were generated at the Johns Hopkins University transgenic core facility. C57BL/6J one-cell embryos (Jackson Laboratories #000664) were injected with Cas9 protein (30 ng/μL), tracrRNA (0·6 μmol/L), crRNA (0·6 μmol/L, see appendix p 9), and ssDNA oligonucleotides (10 ng/μl, see appendix p 9; sourced from Integrated DNA Technologies, Coralville, IA, USA) in buffer (10 mmol/L Tris-HCl pH7·4, 0·25 mmol/L (edetic acid), and transferred to pseudopregnant CD-1 females (Charles River, Wilmington, MA, USA).^18^ Mice were raised in climate and light-controlled conditions with companionship or enrichment. For genotyping, tail DNA was extracted by use of alkaline lysis, amplified and gel extracted (Phusion Flash, QIAquick, Qiagen, Hilden, Germany), and sequenced by use of Big Dye reagents (Thermo Fisher Scientific, 4337455, Waltham, MA, USA) according to manufacturer's protocol (Johns Hopkins Synthesis & Sequencing Facility). Researchers were masked to mouse genotype until data analysis. Mice (11–15 weeks, >seven generations C57BL/6J backcrossed, entire litters) were euthanised (isoflurane, Pennvet VED1360CS, Philadelphia, PA, USA) and underwent intracardiac perfusion (25 mL PBS). After thoracic spinal vertebrae T1–T5 laminectomy, a 23G butterfly needle (Fisher 14-840-35, 4 mm) was inserted into the subarachnoid space and sealed with histoacryl (GoBioMed 10316102, Waterford, MI, USA). 1:10 Methylene Blue:Milli-Q Water (Millipore Sigma, Burlington, MA, USA) was injected at 467 μL/min (Legato 110 pump, Fisher Scientific 14-831-214, Hampton, NH, USA), with pressure monitoring (PX409-100GUSBH, Omega Engineering, Norwalk, CT, USA). Mice were excluded only if a leak was observed at the point of needle insertion due to incomplete sealing. We measured pressure changes on intrathecal infusion of methylene blue-dyed water. A sudden pressure drop or plateau was interpreted as a CSF leak, with the magnitude of pressure drop indicative of either leak number or size. Infused volume (μL), pressure at time of leak (pounds per square inch [psi]), change in pressure during leak (Δpsi), meningeal compliance (ΔμL/Δpsi) and number of mice with leak were assessed. All the above mouse procedures were in accordance with the Johns Hopkins Animal Care and Use Committee (approved ethics protocol number MO24M50) and in adherence to the ARRIVE guidelines.

### Statistical analysis

The gene burden analysis tested case enrichment for rare functional variants by use of Fisher's exact test in all cohorts with false discovery rate correction for multiple comparisons in the Mendel discovery cohort. For this burden analysis, individuals were used as the unit of analysis (rather than alleles) as this is the more stringent test. Observed versus expected domain-specific variant enrichment was compared by χ^2^ test. Human dural fibroblast binding data and mouse biomechanical testing data are presented as mean (SD). For mouse data, comparisons were made between wild-type mice and *Fbn2*^A1052T/+^, *Fbn2*^D1581V/+^, *Fbn2*^M2387T/+^, and *Fbn1*^C1039G/+^ mice (GraphPad Prism version 9, San Diego, CA, USA; unmasked). Mice were bred to detect an effect size of 10% with a power of 80% (goal of 16 mice per group) for biomechanical testing outcomes as above. In-vitro and in-vivo experimental data were compared by use of the global Kruskal–Wallis test, followed by Dunnett comparisons, except for proportions. For in-vitro data, comparisons were made between wild-type and mutant conditions, or uninhibited and inhibited fragments. Proportions of mice with leaks were compared between mutant and wild-type mice by use of χ^2^ tests. p<0·05 was used as significance threshold.

### Role of the funding source

The funders of the study had no role in study design, data collection, data analysis, data interpretation, or writing of the report.

## Results

We did whole-exome sequencing on 42 unrelated individuals with type 1b spontaneous spinal CSF leaks seen as outpatients between Jan 1, 2006 and Dec 31, 2019 (35 [83%] were women, seven [17%] were men, 38 [90%] were White, two [5%] were Black, and two [5%] were Hispanic) and compared these data with whole-exome sequencing data from 2244 controls from the Mendel discovery cohort. 369 genes showed substantial enrichment for rare functional variants in patients with type 1b spontaneous spinal CSF leaks. We prioritised candidates by selecting genes that harboured rare functional variants in more than 10% of individuals with type 1b spontaneous spinal CSF leaks and less than 10% of controls (narrowing to 71 genes) and that showed human dura mater expression (narrowing to 11 genes).^16^ The eleven remaining genes, ranked by the number of individuals with type 1b spontaneous spinal CSF leaks having a rare variant, were *FBN2* (nine [21%] of 42), *AGRN* (eight [19%]), *PARP1* (six [14%]), *HDAC7* (six [14%]), *BAZ1B* (five [12%]), *ZDHHC5* (five [12%]), *PLXNA1* (five [12%]), *ERBB2* (five [12%]), *SNED1* (five [12%]), *KIF1C* (five [12%]), and *ZNF638* (five [12%]). Because *FBN2* variants were the most frequent among individuals with type 1b spontaneous spinal CSF leaks, we prioritised this gene for further variant exploration and functional validation studies. Substantial enrichment in rare functional *FBN2* variants was observed on comparison of the patient cohort to the Mendel discovery cohort (177 [8%] of 2244, p=0·041, odds ratio [OR] 3·18 [95% CI 1·50–6·76]; appendix p 2).

To assess the reproducibility of rare functional *FBN2* variant enrichment, we did burden analyses between the data from the individuals with type 1b spontaneous spinal CSF leaks and two additional validation control cohorts. Substantial enrichment in rare functional *FBN2* variants was also observed in comparison with the Belgian whole-exome cohort (51 [7%] of 714, p=0·004, OR 3·55 [95% CI 1·61–7·81]) and the Belgian thoracic aortic aneurysm and dissection cohort (45 [5%] of 913, p=0·0003, OR 5·26 [95% CI 2·38–11·66]; appendix p 2). The I2394T variant in the TB7 domain of fibrillin-2 occurred in four (5%) of 84 of the alleles of individuals with type 1b spontaneous spinal CSF leaks and at substantially lower frequencies of 0·22–0·49% across gnomAD (663 [0·40%] of 1 613 968) and all control cohorts (appendix p 3), supporting a possible pathogenic role for fibrillin-2 in spontaneous spinal CSF leaks. Of note, one individual with type 1b spontaneous spinal CSF leaks harboured variant I2394T and variant D1588V in heterozygosity.

**Figure 1:**
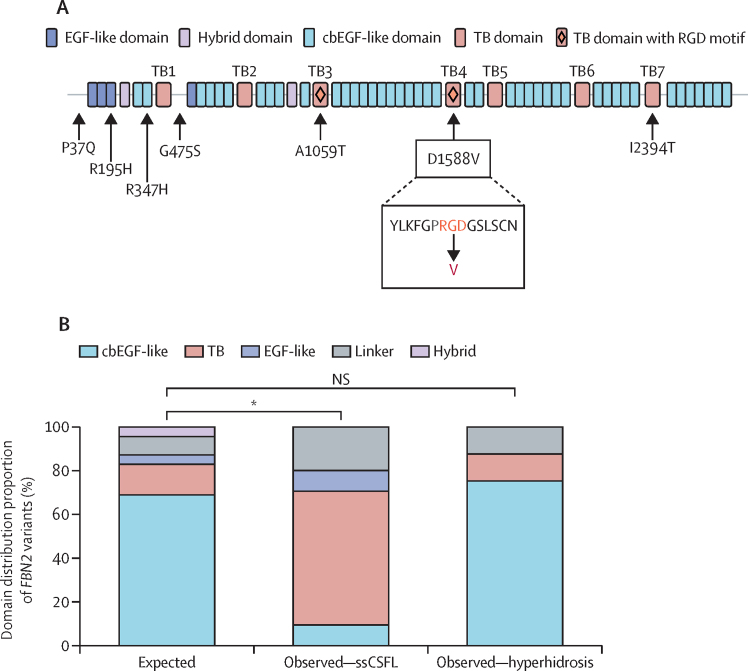
Genetic enrichment and domain-specific localisation of *FBN2* variants in patients with spontaneous spinal CSF leaks (A) Schematic representation of the fibrillin-2 protein showing preferential localisation of rare (MAF<0·01) functional variants in patients with spontaneous spinal CSF leaks (missense, nonsense, frameshift, splicing, and stop-loss) localising to the N-terminal 16% of the protein and TB domains. (B) Proportion of the domain distribution of rare functional *FBN2* variants in patients with spontaneous spinal CSF leaks compared with the expected distribution proportion based on proportional amino acid composition. The proportion of the domain distribution of rare variants in fibrillin-2 associated with hyperhidrosis is included as a negative control. cbEGF=calcium-binding epidermal growth factor. EGF=epidermal growth factor. RGD=arginine–glycine–aspartic acid. ssCSFL=spontaneous spinal CSF leaks. TB=TGF-β binding protein-like. NS=not significant. *p<0·0001.

All patient variants within fibrillin-2 localise to the most N-terminal 16% of the protein or to a TB domain, including one variant in the TB4 RGD domain (D1588V; figure 1A). We found the distribution of variants by domain type to be significantly different from the distribution expected on the basis of random chance: variants were less frequent in cbEGF-like domains (p<0·0001) but enriched in TB domains (p<0·0001; figure 1B). In contrast, hyperhidrosis (a disorder unrelated to connective tissue) displayed a rare variant distribution that did not differ significantly from random chance distribution (p>0·05, figure 1B). With the exception of G475S, all patient variants had combined annotation dependent depletion scores exceeding 20, which indicates that these variants rank among the predicted top 1% most deleterious variants in the human genome, further supporting their potential contribution to disease pathogenesis (table).

**Table:** Frequency of rare *FBN2* variants with CADD scores for each variant

	**Frequency of heterozygosity in individuals with spontaneous spinal CSF leaks**	**Frequency of heterozygosity in gnomAD**	**CADD score**
P37Q	1/42	1·9 × 10^−4^	20·1
R195H	1/42	1·4 × 10^−5^	47
R347H	1/42	5·9 × 10^−3^	24·1
G475S	1/42	3·0 × 10^−5^	12·6
A1059T	1/42	2·5 × 10^−6^	22·2
D1588V	1/42	6·2 × 10^−7^	27·2
I2394T	4/42	4·0 × 10^−3^	22·7

Nearly all *FBN2* variants have CADD scores of more than 20, indicating that they rank among the predicted top 1% most deleterious variants in the human genome. gnomAD=Genome Aggregation Database. CADD=combined annotation dependent depletion.

**Figure 2:**
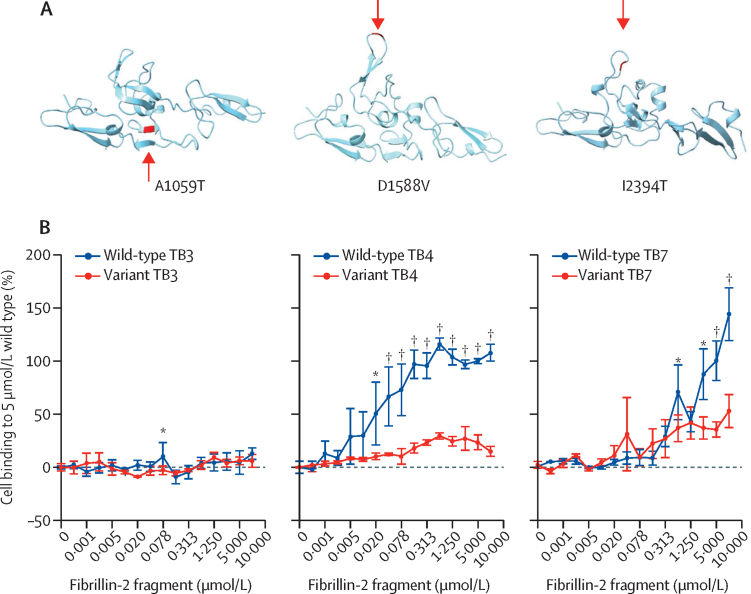
Structural predictions and cell binding properties of fibrillin-2 TB domains with and without spontaneous spinal CSF leak-associated variants (A) AlphaFold2 structural modelling of fibrillin-2 TB domains with neighbouring cbEGF-like domains in their tertiary structure. The locations of patient variants are indicated in red. The RGD motif in TB4, at the location of the D1588V variant, is presented on an accessible flexible loop. The TB7 I2394T variant is similarly located on an accessible flexible loop, whereas the TB3 A1059T variant resides within a β-strand, an inflexible internal region predicted to be inaccessible to cell-surface integrins. (B) Human dural fibroblast binding to recombinant fibrillin-2 TB domain fragments with or without patient variants. Data represent the mean (SD) from three independent experiments with three technical replicates per experiment. cbEGF=calcium-binding epidermal growth factor. RGD=arginine–glycine–aspartic acid. TB=TGF-β binding protein-like.

Although the RGD sequence is the canonical integrin binding site, non-canonical binding sites exist in extracellular matrix proteins. Non-canonical and RGD binding sites can share common structural features. As predicted by AlphaFold2 artificial intelligence modelling, both the TB4 RGD domain variant D1588V and the TB7 variant I2394T are positioned on an easily accessible loop (figure 2A). In contrast, the TB3 domain variant A1059T resides in an internal region and would not be predicted to be accessible to cell-surface integrins.

**Figure 3:**
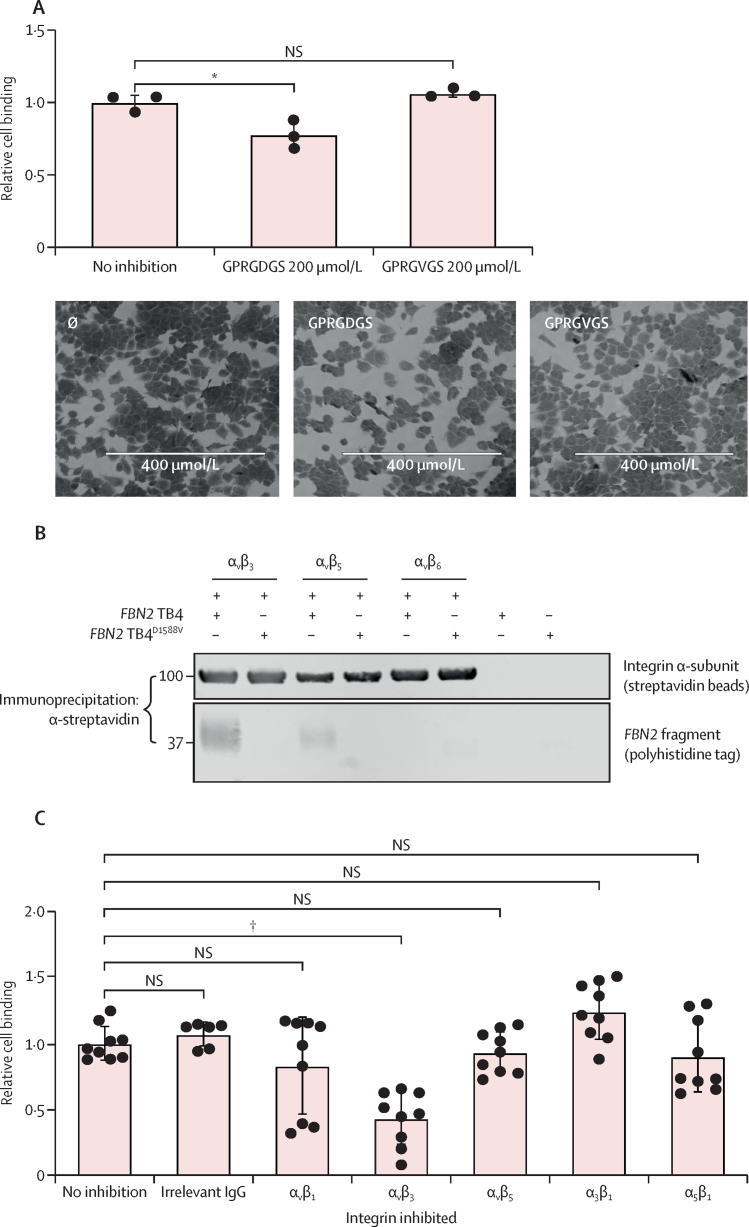
Mechanistic characterisation of fibrillin-2 TB4 domain integrin binding (A) Competitive inhibition of human dural fibroblast binding to wild-type TB4 fibrillin-2 fragments, including quantification of cell binding and representative images. (B) Co-immunoprecipitation assays between fibrillin-2 TB4 fragments and integrin headpieces. (C) Integrin-specific inhibition of human dural fibroblast binding to TB4 fragments. *p<0·001. †p<0·0001.

To test whether cells in the meninges are capable of binding to the TB4 and TB7 domains of fibrillin-2, we recombinantly expressed fibrillin-2 fragments encompassing the TB3, TB4, and TB7 domains (appendix p 4). Binding assays revealed that normal human dural fibroblasts exhibited dose-dependent adhesion to wild-type TB4 fibrillin-2 fragments. This interaction was almost completely abrogated by the D1588V variant (p<0·0001 at 5 mmol/L; figure 2B). Normal human dural fibroblasts also bound the wild-type TB7 fibrillin-2 fragment. This interaction was markedly diminished by I2394T variant (p<0·0001 at 5 mmol/L; figure 2B). In contrast, no detectable binding occurred with the TB3 domain, confirming our prediction based on structural modelling (figure 2B). To confirm that the observed binding defect with the TB4 D1588V variant resulted from the RGD domain dysfunction, we competitively inhibited wild-type TB4 binding using synthetic peptides. We found that preincubation of human dural fibroblasts with GPRGDGS peptide, but not GPRGVGS peptide containing the variant, significantly reduced binding to wild-type TB4 fibrillin-2 fragments (p=0·0050; figure 3A).

To identify which integrins bind the TB4 and TB7 fibrillin-2 domains, we did co-immunoprecipitation assays between fibrillin-2 fragments and a_v_b_3_, a_v_b_5_ or a_v_b_6_ integrin headpieces. These experiments showed robust binding of wild-type TB4 fibrillin-2 fragments to α_v_β_3_, weaker binding to α_v_β_5,_ and no binding to α_v_β_6_ (figure 3B). Neither mutant TB4 fragments, nor wild-type or mutant TB3 fragments, bound any tested integrins (figure 3B; appendix p 5). All TB7 fragments non-specifically bound streptavidin beads (appendix p 5), precluding reliable assessment of TB7-integrin interactions using this approach. We validated these findings using human dural fibroblast binding assays. Fibroblast binding to the TB4 fragment was significantly impaired by α_v_β_3_ integrin inhibition (p<0·0001), but remained unaffected by α_v_β_1_, α_v_β_5_, α_3_β_1_, or α_5_β_1_ inhibition (figure 3C). Contrastingly, human dural fibroblast binding to the TB7 fragment was impaired by α_v_β_3_ or α_v_β_5_ inhibition (p<0·01), but not by α_v_β_1_, α_3_β_1_, or α_5_β_1_ inhibition (appendix p 5). We did competitive binding assays to test whether integrins bind TB7 through the same binding pocket used for RGD motif recognition. We found that GPRGDGS peptide, but not GPRGVGS peptide, selectively inhibited fibroblast binding to TB7 (appendix p 5), suggesting that integrins use a common binding mechanism for TB4 and TB7.

**Figure 4:**
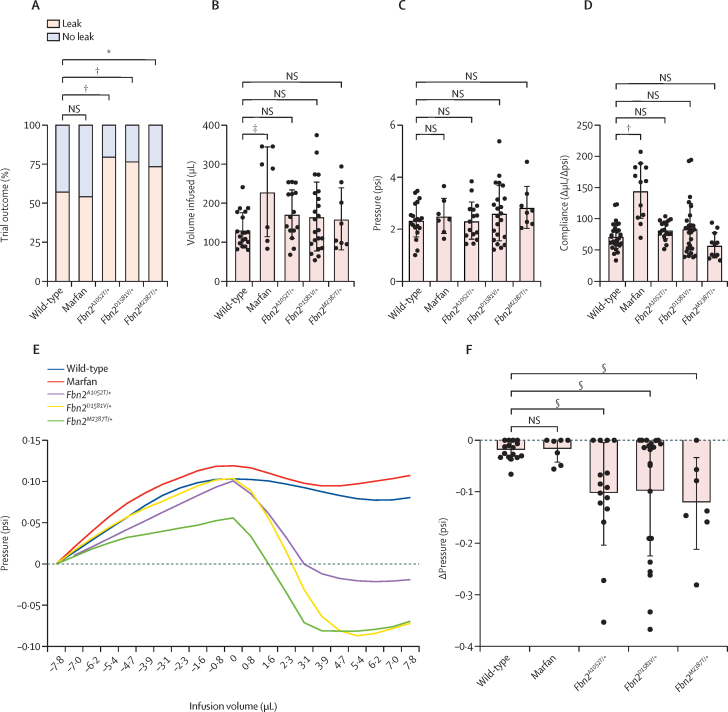
In vivo functional assessment of *Fbn2* variants associated with spontaneous spinal CSF leaks and Marfan variant in mouse models (A) Proportion of CSF leak occurrence in mouse strains following intrathecal infusion. Leaks were defined as a sudden drop or plateau in pressure during intrathecal infusion trial. (B) Volume of methylene blue solution required to initiate CSF leak in mice in which a leak occurred. *Fbn2* mutant mice require similar volumes to wild-type controls, whereas Marfan syndrome mice require significantly larger volumes. (C) Pressure at leak initiation shows no significant differences between any mutant strain and wild-type mice. (D) Meningeal compliance (ΔμL/Δpsi) calculated from pressure–volume curve slopes before leak onset. Marfan syndrome mice alone show significantly increased compliance relative to wild-type controls. (E) Representative pressure traces during intrathecal infusion trials showing characteristic pressure drop patterns at leak initiation for each genotype. *Fbn2* mutants exhibit more dramatic pressure drops compared with wild-type and Marfan controls. (F) Magnitude of pressure drop at the time of CSF leak. All *Fbn2* mutant mice show significantly larger pressure drops compared with both wild-type mice. Wild-type and Marfan syndrome mice do not differ from each other. NS=not significant. psi=pounds per square inch. *p<0·001. †p<0·0001. ‡p<0·01. §p<0·05.

To establish whether variants associated with type 1b spontaneous spinal CSF leaks cause functional impairment in vivo, we did CSF leak experiments in mice carrying variants equivalent to each of the fibrillin-2 TB domain variants found in patients with type 1b spontaneous spinal CSF leaks (ie, A1059T, D1588V and I2394T; figure 1A). *Fbn2*^A1052T/+^, *Fbn2*^D1581V/+^, and *Fbn2*^M2387T/+^ mice were also compared with Marfan syndrome mice (*Fbn1*^C1039G/+^), as patients with Marfan syndrome have dural pathology including dural ectasia and increased spontaneous spinal CSF leak prevalence. There was no significant increase in CSF leak frequency in Marfan syndrome mice compared with wild-type mice (seven [54%] of 13 *vs* 20 [57%] of 35, respectively, p>0·05, relative risk [RR]=0·94, 95% CI 0·74–1·22, figure 4A). Contrastingly, these frequencies were significantly higher for *Fbn2*^A1052T/+^ mice (15 [79%] of 19), RR 1·38, 95% CI 1·14–1·69), for *Fbn2*^D1581V/+^ mice (22 [76%] of 29], RR 1·33, 95% CI 1·09–1·63), and for *Fbn2*^M2387T/+^ mice (eight [73%] of 11], RR 1·27, 95% CI 1·04–1·58), with all comparisons p<0·001 (figure 4A).

Furthermore, mice harbouring *Fbn2* variants had CSF leaks after infusion of similar volumes and at similar pressures when compared with wild-type mice (p>0·05; figure 4B–C). In contrast, Marfan syndrome mice required significantly larger infusion volumes to initiate leakage (p=0·01; figure 4b), although leak initiation occurred at similar pressures (figure 4C). We calculated meningeal compliance from the slopes of stress–strain curves measured before CSF leak onset. Only Marfan syndrome mice showed significantly increased compliance relative to wild-type controls (*p*=0·0002; figure 4D). Notably, all mice harbouring *Fbn2* variants showed a significantly larger pressure drop at the time of leak compared with wild-type or Marfan syndrome mice (all p<0·05; figure 4E–F), which did not differ from each other.

## Discussion

We provided compelling evidence that rare deleterious *FBN2* variants are a major genetic risk factor for type 1b spontaneous spinal CSF leaks. We did functional validation of the *FBN2* variants and showed the TB4 and TB7 domains of fibrillin-2 harbouring patient variants lose human dural fibroblast binding ability. We also showed increased CSF leak susceptibility and severity in fibrillin-2 mutant mice, and increased dural compliance in Marfan syndrome mice.

Our findings expand the phenotypic spectrum of fibrillin-2-related heritable connective tissue disorders. Mutations in fibrillin-2 cbEGF domains have previously been identified in patients with congenital contractural arachnodactyly who do not typically develop spontaneous spinal CSF leaks. Reports have also identified fibrillin-2 mutations as causative of carpal tunnel syndrome,^15^ including functional mutations in cbEGF domains. Two variants (R347H and I2394T) identified in our study in patients with type 1b spontaneous spinal CSF leaks were also reported in carpal tunnel syndrome,^15^ suggesting potentially shared mechanistic underpinnings.

Our exclusion of patients with inciting trauma was intended to enrich for individuals with high genetic predisposition to leak development. However, approximately one-third of patients with spontaneous spinal CSF leaks have precipitation of mildly traumatic events, such as chiropractic spinal adjustment, indicating substantial environmental contribution and supporting gene–environment interactions with disease pathogenesis.^7^ The frequency of *FBN2* variants is also non-negligible in control populations. Together, these data suggest that *FBN2* variants predispose to type 1b spontaneous spinal CSF leaks with incomplete penetrance.

All type 1b spontaneous spinal CSF leak variants outside of the TB domains localise to the N-terminal of fibrillin-2, which is important for the beaded microfibril assembly required for fibrillin function. It remains to be shown whether N-terminal variants directly affect cell–matrix interactions.

Binding of α_v_β_3_ integrin to fibrillin-2 TB4 through its RGD sequence was previously established.^19^ Our work confirms and extends these findings by showing relevance to human dural fibroblasts in a disease context. We identified TB7-mediated cell binding through α_v_β_3_ and α_v_β_5_ integrins. We found no evidence of dural fibroblast binding to the mammalian-expressed TB3 domain. Previous studies have inconsistently reported cell adhesion to fibrillin-2 TB3. These inconsistent findings could be caused by expression system differences (bacterial *vs* mammalian cell).^10^, ^20^

Our findings revealed that type 1b spontaneous spinal CSF leak-associated variants in the TB3, TB4, and TB7 domains of fibrillin-2 led to increased CSF leak frequency and severity in mice, a characteristic not attributable to increased pressure or volume at the time of leak. Contrastingly, in Marfan syndrome mice, larger infusion volumes were required to induce leak. Marfan syndrome mice also showed increased meningeal compliance without increased leak frequency or severity. Across all mouse strains tested, increased pressure was not associated with leak predisposition. Whereas most patients with Marfan syndrome develop dural ectasia,^21^ which is the development of lumbar spinal dura outpouchings,^22^ patients with spontaneous spinal CSF leaks do not show increased propensity for dural ectasia. The increased dural compliance observed in Marfan syndrome mice might correspond to the dural ectasia observed in patients with Marfan syndrome. The pressure-independent and volume-independent leak susceptibility in *Fbn2* variant mice suggests an alternative pathophysiological pathway. These observations collectively suggest that spontaneous spinal CSF leaks in patients with Marfan syndrome probably operates through a different mechanism from spontaneous spinal CSF leaks in patients with rare *FBN2* variants. Neither mechanism appears to be driven by increased intrathecal pressure.

Our study has limitations. First, in the genetic study, our validation control cohorts were drawn from the Belgian population whereas the affected individuals were admixed Americans. Although population-specific allele frequencies could theoretically contribute to apparent differences between cases and controls (ie, population stratification), this concern is minimised by the exclusive focus on rare and predicted deleterious alleles in both groups. Both cases and controls were largely of Caucasian European ancestry—potentially limiting the generalisability of our findings to other ethnicities. Second, the generated mouse models also have limitations. The amino acid affected by the type 1b spontaneous spinal CSF leaks-associated variant, I2394T, is not conserved between humans and mice. However, isoleucine (an amino acid in the human sequence) and methionine (an amino acid in the mouse sequence) are both hydrophobic residues. Their substitution with a polar residue (threonine) is likely to have similar functional consequences. Furthermore, important anatomical differences exist between mice and humans, with mouse dura being proportionally thinner.^23^ The quadrupedal posture in mice eliminates the increased hydrostatic pressure that is characteristic of human lower spinal regions. Furthermore, our rapid leak induction protocol cannot recapitulate the gradual compromise of spinal meningeal integrity that probably occurs over decades in human patients, given that spontaneous spinal CSF leaks typically manifest in middle age.

No pharmacological treatments exist for spontaneous spinal CSF leaks. Previous work in our laboratory suggested that manifestations of connective tissue diseases can be treatable through targeted pharmacological interventions rather than solely through surgical interventions.^24^, ^25^ Given the substantial burden and risks associated with current treatments, development of drug therapies addressing the molecular causes of this condition is a high-priority research direction.

This study advances our understanding of type 1b spontaneous spinal CSF leaks’ pathogenesis by identifying *FBN2* as the first gene associated with disease susceptibility and establishing experimental models for future therapeutic development. Our findings transform type 1b spontaneous spinal CSF leaks from an idiopathic condition to one with a defined genetic underpinning within the fibrillinopathy spectrum, and provide a foundation for improved diagnosis and targeted treatment development.

## Contributors

## Data sharing

Deidentified data will be shared after reasonable request from investigators within 5 years of publication. Proposals for experimental data should be directed to cparks21@jhmi.edu, and proposals for human genomic data should be directed to nsobrei2@jhmi.edu. Links will then be provided for data access at a third-party website.

## Declaration of interests

The authors declare no competing interests.
