# Supplementary material for: Spontaneous spinal CSF leaks: a rare variant exome sequencing study and functional analysis
Source: Lancet Neurol. 2026 Jul;25(7):664–72. doi: 10.1016/S1474-4422(26)00140-7 (PMC13275636; doi:10.1016/S1474-4422(26)00140-7)
Supplement: Supplementary appendix [file mmc1.pdf]

THE LANCET
Neurology

Supplementary appendix

This appendix formed part of the original submission and has been peer reviewed. We post it as supplied by the authors.

Supplement to: Parks CA, Singh M, Wohler E, et al. Spontaneous spinal CSF leaks: a rare variant exome sequencing study and functional analysis. *Lancet Neurol* 2026; **25:** 664–72.

Supplementary methods
Functional analysis

To generate in-silico models of fibrillin-2 TGFβ-binding protein-like (TB) domain fragments (UniProt ID P35556), we used AlphaFold v2.1.1 (Google DeepMind, United Kingdom)[1] and visualized them in ChimeraX (UCSF, United States of America).[2]

For functional analyses on recombinant peptides, wild-type and mutant TB3, TB4, and TB7 regions of fibrillin-2 (UniProt ID P35556) with flanking calcium binding epidermal growth factor-like (cbEGF-like) domains (two domains N- and C-terminal for TB4/TB7; one N-terminal and two C-terminal for TB3) were synthesized by cloning mammalian codon-optimized gene fragments into pHLSec[3] (Addgene, 99845)/pCDH vectors (Addgene, 72265) amplified using C3019 bacteria (New England Biosciences, USA), expressed using Expi293 cells (ThermoFisher, A14635), and purified as previously described.[4,5] 96-well plates were coated with wild-type or mutant TB3, TB4, or TB7 fibrillin-2 fragments in serial dilution for binding studies, or at 5μM for peptide/anti-integrin antibody inhibition experiments. Passage 2 Human Dural Fibroblasts (ScienCell #1420) were seeded (1×10^5 cells/well) after pre-incubation (10min, 37°C) with: GPRGDGS or control (GPRGVGS) peptides (ThermoFisher MDB103253) to evaluate RGD-dependent binding, or antibodies (10μg/mL) against integrins αvβ1 (Thermo Fisher Scientific, BS-2016R), αvβ3 [LM609, AbCam ab190147], αvβ5 [P1F6, AbCam ab177004], α3β1 [M-KID2, Fisher Scientific MAB1922MI], or α5β1 [Jbs5, Thermo Fisher Scientific BS-2016R] to evaluate the integrin repertoire used by human dural fibroblasts to bind to fibrillin-2. Binding between human dural fibroblasts and fibrillin-2 fragments was quantified with crystal violet staining, cell permeabilization, and 595nm absorbance.[6] Cell binding data were normalized to wild-type TB4 binding at 5mM with average BSA control absorbance subtracted. 2μg of wild-type and mutant TB3, TB4, and TB7 fibrillin-2 fragments were incubated with 1μg of integrin αvβ3, αvβ5, or αvβ6 recombinant headpieces (expressed and purified as previously described in Godwin et al. (2023)[7]) in 20mM 4-(2-hydroxyethyl)-1-piperazineethanesulfonic acid (HEPES) buffer, 150mM NaCl, 0.5mM Ca^{2+}/Mg^{2+}/Mn^{2+}, 0.05% Tween-20, pH7.4 for 2h at room temperature, captured using MagStrep® Strep-Tactin®XT beads (2h, RT), washed thrice with HEPES buffer, and eluted in NuPAGE LDS sample buffer (Thermo Fisher Scientific) containing 20% β-mercaptoethanol. Western blotting used anti-6-His (Bethyl, 1:5000) and anti-strep (IBA, 1:5000) primary antibodies to probe for fibrillin-2 or integrin headpiece fragments, respectively, with IRDye® secondary antibodies (LiCorBio, 1:10,000).

Table 1.

Cohort name	$n_{individuals\ with\ FBN2\ variants}$ / $n_{individuals\ in\ cohort}$	*p*-value	OR (95% CI)
ssCSFL cases	9/42	N/A	N/A
Mendel Initiative controls (USA)	177/2244	0.0407	3.18 (1.50-6.76)
Whole-exome controls (Belgium)	51/714	0.004	3.55 (1.61-7.81)
TAAD controls (Belgium)	45/913	<0.001	5.26 (2.38-11.66)

Table 1. Burden analysis showing significant enrichment of rare functional *FBN2* variants in ssCSFL patients compared to three independent control cohorts.

Table 2

Cohort Name	I2394T Allele Frequency (n/N)	*p*-value
ssCSFL cases	0.048 (4/84)	N/A
Mendel Initiative controls (USA)	0.0048 (21/4488)	0.044
Whole-exome controls (Belgium)	0.0049 (7/1428)	0.004
TAAD controls (Belgium)	0.0022 (4/1826)	<0.001

Table 2. Allele frequency comparison for the p.I2394T variant in TB7 domain across populations. The variant shows striking enrichment in ssCSFL probands (4·8% of alleles) compared to substantially lower frequencies in gnomAD (0·40%, 663/1613968) and control cohorts (0·22-0·49%).

Supplementary Figure 1.

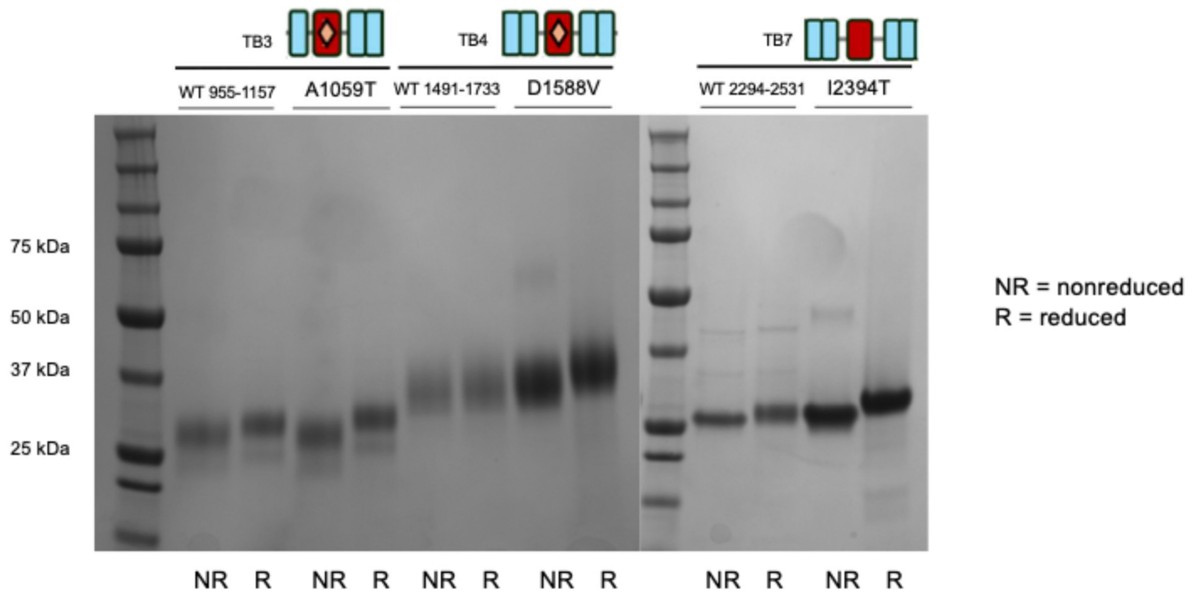

Supplementary Figure 1. SDS-PAGE gel electrophoresis of recombinant fibrillin-2 TB3, TB4, and TB7 domain fragments under reducing and non-reducing conditions. Includes diagrams above relevant lanes depicting expressed cbEGF-like domains. The difference in migration patterns between reduced and non-reduced samples demonstrates successful formation of disulfide bond-dependent secondary structure in mammalian-expressed fragments. Molecular weight markers are shown on the left.

Supplementary Figure 2.

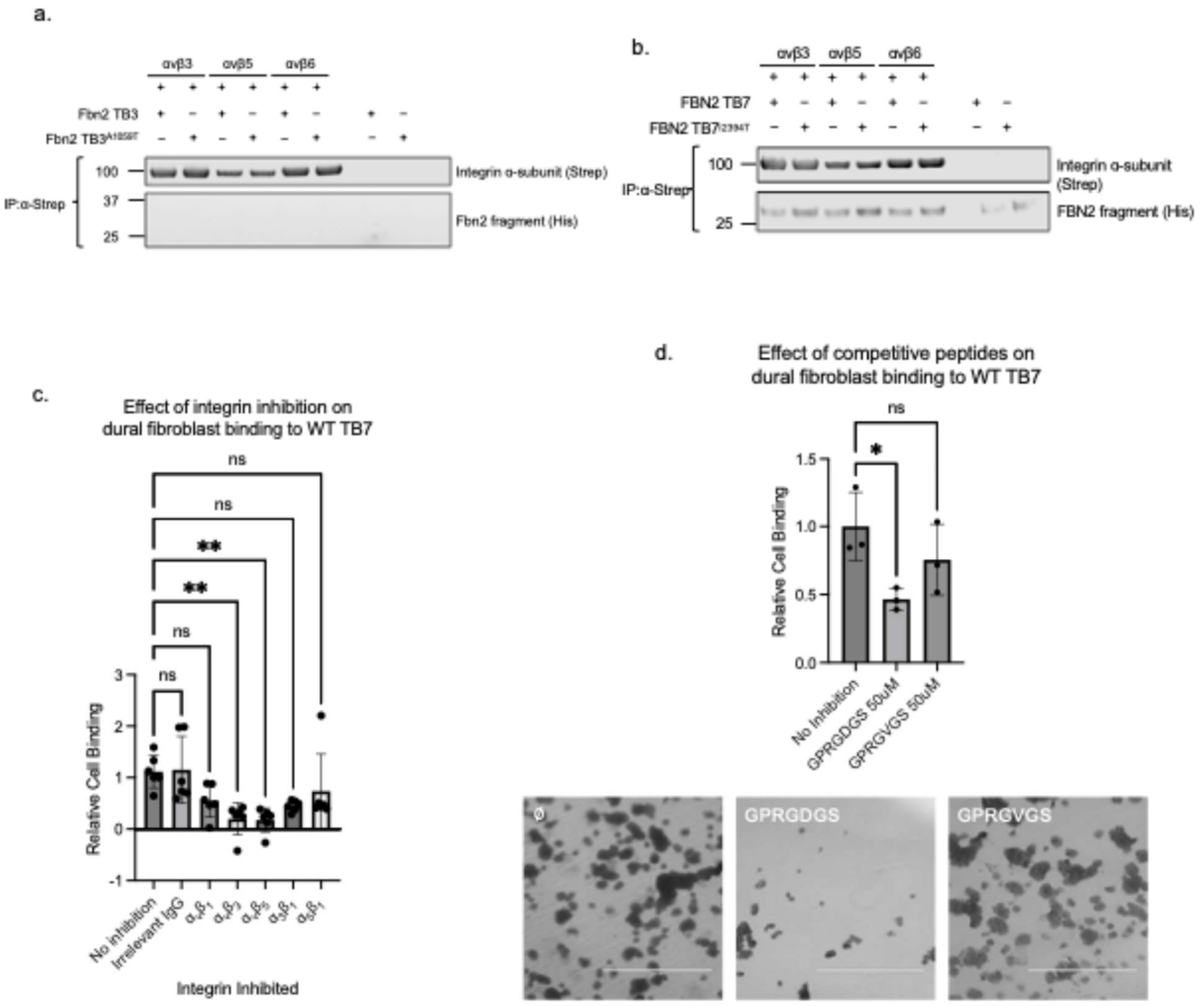

Supplementary Figure 2. Assessment of fibrillin-2 domain TB3 and TB7 fragment binding to αvβ-integrins. a,b. Co-immunoprecipitation assays showing the amount of a. TB3:His and TB3^{A1059T}:His, b.TB7:His and TB7^{I2394T}:His proteins immunoprecipitated with αvβ3:Strep, αvβ5:Strep and αvβ6:Strep integrins immobilised on anti-Strep matrix. Protein-protein interactions were resolved using SDS-PAGE and immunodetected using anti-His and anti-Strep antibodies. TB3 and TB3^{A1059T} do not interact with integrins. TB7 and TB7^{I2394T} show nonspecific binding to the anti-Strep matrix. (n=4) **c.** Integrin-specific inhibition of human dural fibroblast binding to wild-type TB7 fibrillin-2 fragments (plates coated at 20uM). Cell binding is significantly impaired by inhibition of both αvβ3 ($p = 0.0042$) and αvβ5 integrins ($p = 0.0033$) but remains unaffected by inhibition of αvβ1, α3β1, or α5β1, indicating that TB7-mediated cell adhesion occurs through multiple integrin heterodimers. d. Competitive inhibition of human dural fibroblast binding to wild-

type TB7 fibrillin-2 fragments, including quantification of cell binding and representative images (scale bar = 400μm). Pre-incubation with GPRGDGS peptides at 50uM significantly reduces cell binding ($p = 0.0022$), while using mutant GPRGVGS peptides has no effect, confirming RGD motif-dependent binding. Data represent mean ± SD.

Supplementary Figure 3.

Species	A1059T variant	D1588V variant	I2394T variant
Human *(Homo sapiens)*	GAGFANRGDVLTG	KFGPRGDGSLS	VLQTICQMAS
Western lowland gorilla *(Gorilla gorilla gorilla)*	GAGFANRGDVLTG	KFGPRGDGSLS	VLQTICQMAS
Crab-eating macaque *(Macaca fascicularis)*	GAGFANRGDVLTG	KFGPRGDGSLS	VLQTICQMAS
Asian Tiger *(Panthera tigris)*	GPGFANRGDVLTG	KFGPRGDGSLS	VLQTMCQMAS
Greater spear-nosed bat *(Phyllostomus bastatus)*	GPGFANRGDVLTG	KFGPRGDGSLS	VLQTMCQMAS
Southern elephant seal *(Mirounga leonina)*	GPGFANRGDVLTG	KFGPRGDGSLS	VLQTMCQMAS
Sperm whale *(Physeter catodon)*	GPGFANRGDVLTG	KFGPRGDGSLS	VLQTMCQMAS
Wild Bactrian Camel *(Camelus ferus)*	GPGFANRGDVLTG	KFGPRGDGSLS	VLQTMCQMAS
Common warthog *(Phacochoerus africanus)*	GPGFANRGDVLTG	KFGPRGDGSLS	VLQTMCQMAS
House Mouse *(Mus musculus)*	GPGFANRGDILTG	KFGPRGDGSLS	VLQTMCQMAS
Red-clawed frog *(Xenoups tropicalis)*	GPGFTNRGDVLSG	NHGARGDGTIS	VLQTMCQMAS
Aeolian wall lizard *(Podarcis raffonei)*	GPGFSNRGDILTG	KYGPRGDGSLS	VLQTMCQMAS
Mississippi paddlefish *(Polydon spathula)*	GPGFANRGDILTG	EFGYRGDGSLA	VLQTMCQMAS
Zebrafish *(Danio reiro)*	GPGFANKGDVLTG	DTLDRGDGGIS	VLQTMCQMAS
Elephant Shark *(Callorhinchus milii)*	GPGFANRGDILTG	EHSTRGDGSLS	VLQTMCQMSS
Common limpet sea snail *(Patella vulgata)*	-----------	APDIRGDVNEC	-----------

Location of patient variant

Deviation from human sequence

Supplementary Figure 3. Evolutionary conservation of fibrillin-2 mutation sites across species. Multiple sequence alignment showing evolutionary conservation of amino acid residues corresponding to ssCSFL patient mutation sites across vertebrate species. The TB3 mutation position (p.A1059T) shows conservation in the majority of species, with exceptions in amphibians and reptiles. The TB4 mutation position (p.D1588V) demonstrates absolute conservation of the aspartic acid residue across species. The TB7 mutation position (p.I2394T) is conserved as either isoleucine (humans) or methionine (mice and other species), both hydrophobic residues. Non-conserved residues are highlighted in red, and mutation positions are indicated in yellow.

Supplementary Figure 4

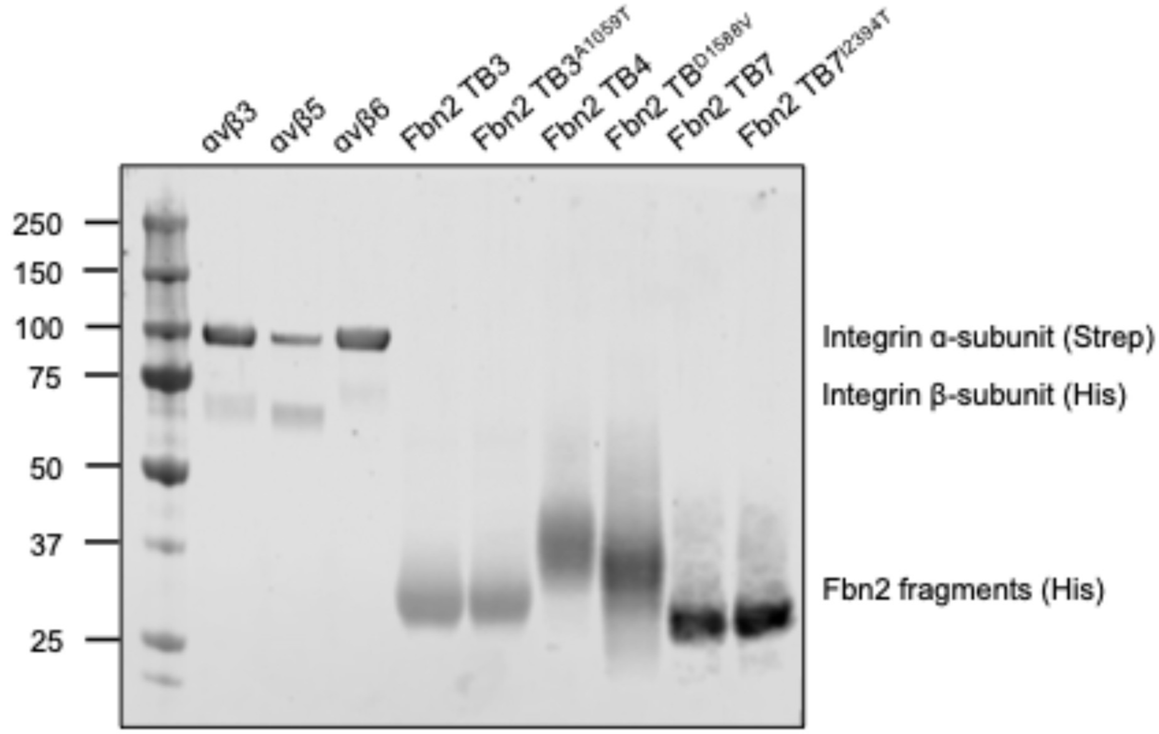

Supplementary Figure 4. Fibrillin-2 domain fragments and integrin input samples used in co-immunoprecipitation assays.

Western blot showing the relative expression levels of recombinant integrin, and wild-type and mutant fibrillin-2 proteins. Proteins were resolved using SDS-PAGE and immunodetected using anti-His and anti-Strep antibodies. (n=4)

Supplementary Figure 5.

crRNA sequences:
A1052T: GGCCCAGGCTTTGCTAACAG

D1581V: GACAGACTCCCGTCTCCTC

M2387T: ACTGCAGACAATGTGTCAGA

Oligos:
A1052T:
GGCACCAAGGAATATGAGACCCTGTGCCCGCGGGGCCCAGGGTTTACTAACAGAG
GGGACATCCTCACTGGGCGTCCATTTTACAAAGGTAAGTGAGTAC

D1581V:
CGACTGACACCTACGCCGACCTCTGTGTTGCAGGAGAGACTCCCGACTCCTCGAG
GACCAAACTTCAGGTAACAGTTGCCCACGCGGTTGTCTGCAGAGC

M2387T:
AGACAACCGCCAGGGTCTGTGCTTCGCCGAGGTACTGCAAACAACATGTCAGATG
GCCTCCAGTAGCCGCAACCTCGTCACAAAGTCGGAGTGCTGCTGT

Supplementary Figure 5. crRNA sequences and oligonucleotide sequences used for Crispr-Cas9 generation of fibrillin-2 mutant mice.

Supplementary Figure 6

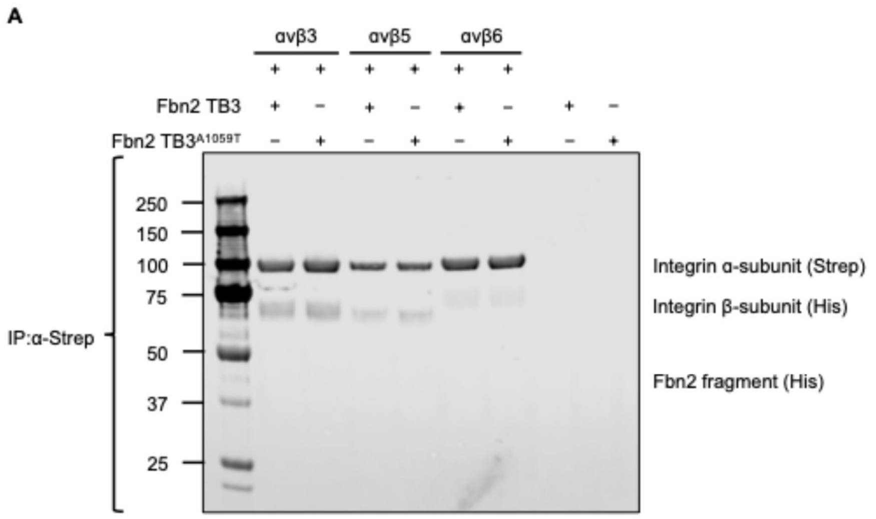

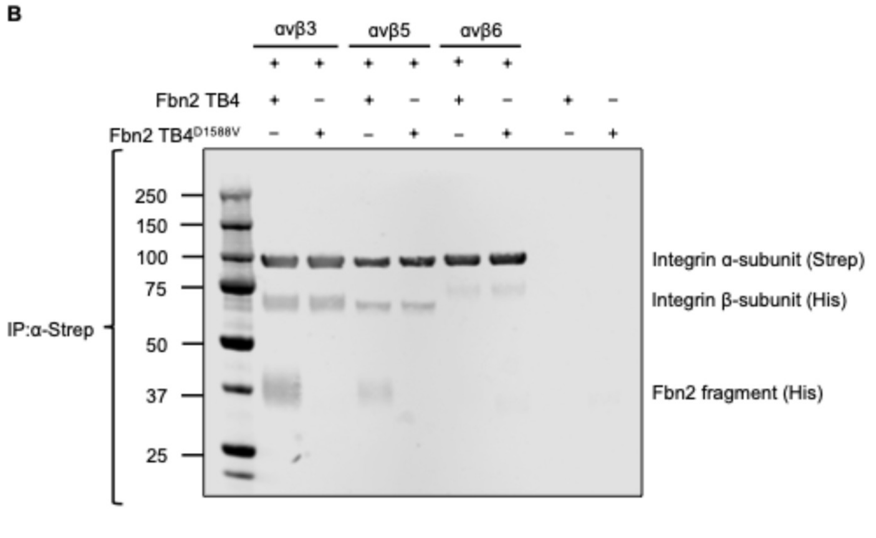

c

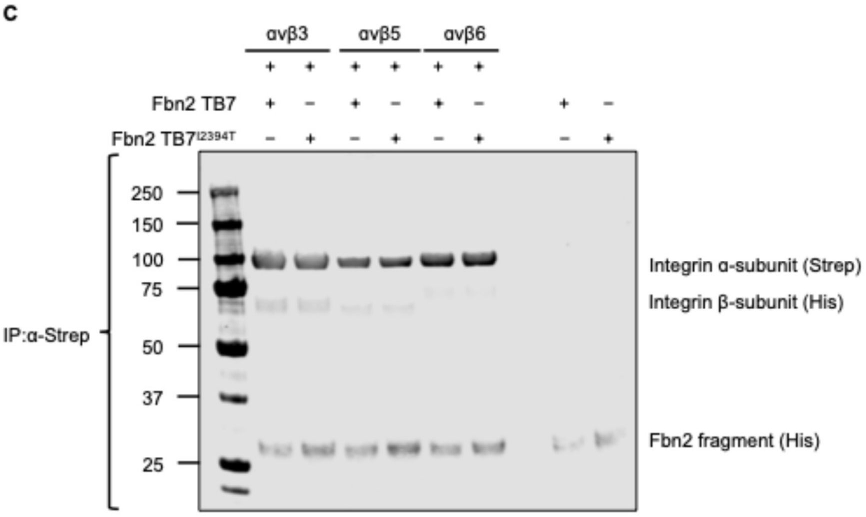

Supplementary Figure 6. Full, uncropped western blots from co-IP experiments

Uncropped western blots corresponding to **a.** Supplementary Figure 2a, **b.** Figure 3b, **c.** Supplementary Figure 2b co-IP experiments.

1. Jumper J, Evans R, Pritzel A, Green T, Figurnov M, Ronneberger O, et al. Highly accurate protein structure prediction with AlphaFold. Nature. 2021 Aug;596(7873):583–9.

2. Pettersen EF, Goddard TD, Huang CC, Meng EC, Couch GS, Croll TI, et al. UCSF ChimeraX: Structure visualization for researchers, educators, and developers. Protein Sci. 2021 Jan;30(1):70–82.

3. Aricescu AR, Lu W, Jones EY. A time- and cost-efficient system for high-level protein production in mammalian cells. Acta Crystallogr D Biol Crystallogr. 2006 Oct;62(Pt 10):1243–50.

4. Singh M, Baldock C. Purification of Recombinant ADAMTSL2. Methods Mol Biol. 2020;2043:157–72.

5. Singh M, Cain SA, Baldock C. Molecular Cloning, Lentiviral Transduction, and Expression of Recombinant ADAMTSL2 and ADAMTSL4. In: Apte SS, editor. ADAMTS Proteases: Methods and Protocols [Internet]. New York, NY: Springer; 2020 [cited 2025 May 27]. p. 137–55. Available from: https://doi.org/10.1007/978-1-4939-9698-8_12

6. Del Cid JS, Reed NI, Molnar K, Liu S, Dang B, Jensen SA, et al. A disease-associated mutation in fibrillin-1 differentially regulates integrin-mediated cell adhesion. J Biol Chem. 2019 Nov 29;294(48):18232–43.

7. Godwin ARF, Dajani R, Zhang X, Thomson J, Holmes DF, Adamo CS, et al. Fibrillin microfibril structure identifies long-range effects of inherited pathogenic mutations affecting a key regulatory latent TGFβ-binding site. Nat Struct Mol Biol. 2023;30(5):608–18.
